# Postoperative prolonged mechanical ventilation correlates to poor survival in patients with surgically treated spinal metastasis

**DOI:** 10.3389/fonc.2022.940790

**Published:** 2022-10-27

**Authors:** Motaz Hamed, Simon Brandecker, Shaleen Rana, Anna-Laura Potthoff, Lars Eichhorn, Christian Bode, Frederic Carsten Schmeel, Alexander Radbruch, Niklas Schäfer, Ulrich Herrlinger, Mümtaz Köksal, Frank Anton Giordano, Hartmut Vatter, Matthias Schneider, Mohammed Banat

**Affiliations:** ^1^ Department of Neurosurgery, University Hospital Bonn, Bonn, Germany; ^2^ Department of Anesthesiology and Intensive Care Medicine, University Hospital Bonn, Bonn, Germany; ^3^ Department of Neuroradiology, University Hospital Bonn, Bonn, Germany; ^4^ Division of Clinical Neurooncology, Department of Neurology, University Hospital Bonn, Bonn, Germany; ^5^ Department of Radiation Oncology, University Hospital Bonn, Bonn, Germany

**Keywords:** prolonged mechanical ventilation (PMV), spinal metastasis treatment, spinal surgery, spinal surgery and complications, mortality

## Abstract

**Objective:**

Patients with spinal metastasis (SM) are at advanced stages of systemic cancer disease. Surgical therapy for SM is a common treatment modality enabling histopathological diagnosis and the prevention of severe neurological deficits. However, surgery for SM in this vulnerable patient cohort may require prolonged postoperative intensive care treatment, which could adversely affect the anticipated benefit of the surgery. We therefore assessed postoperative prolonged mechanical ventilation (PMV) as an indicator for intensive care treatment with regard to potential correlations with early postoperative mortality and overall survival (OS).

**Methods:**

Between 2015 and 2019, 198 patients were surgically treated for SM at the author´s neurosurgical department. PMV was defined as postoperative mechanical ventilation of more than 24 hours. A multivariate analysis was performed to identify pre- and perioperative collectable predictors for 30 days mortality.

**Results:**

Twenty out of 198 patients (10%) with SM suffered from postoperative PMV. Patients with PMV exhibited a median OS rate of 1 month compared to 12 months for patients without PMV (p < 0.0001). The 30 days mortality was 70% and after one year 100%. The multivariate analysis identified “PMV > 24 hrs” (p < 0.001, OR 0.3, 95% CI 0.02-0.4) as the only significant and independent predictor for 30 days mortality (Nagelkerke’s R2 0.38).

**Conclusions:**

Our data indicate postoperative PMV to significantly correlate to high early postoperative mortality rates as well as to poor OS in patients with surgically treated SM. These findings might encourage the initiation of further multicenter studies to comprehensively investigate PMV as a so far underestimated negative prognostic factor in the course of surgical treatment for SM.

## Introduction

Tumor disease with spinal metastases (SM) plays an increasing role in daily clinical practice (1) and surgery is a common treatment option for this highly affected patient cohort (2). Along with the lung and the liver, the skeletal system is among the most common locations of systemic metastasis (3, 4). Surgical treatment options comprise biopsy with vertebroplasty/kyphoplasty (5), decompression alone (6), or decompression in combination with percutaneous (7) or open instrumentation (8, 9). The goal of surgery is to minimize or prevent neurological deficits and to improve the patient*’*s quality of life (10). The indication for surgery must be interdisciplinary, taking into account the urgency, the therapeutic objective, aspects of stability of spinal biomechanics and prognostic considerations of the underlying conditions (11, 12). Different score systems for estimating the prognosis and survival have limited capacity and can only be used as guide points (13, 14). Nevertheless, surgical treatment may require prolonged postoperative intensive care, which could adversely affect the anticipated benefits of the surgery. Postoperative prolonged mechanical ventilation (PMV) has recently been used as an indicator variable for intensive care treatment in several diseases (15–18). However, the impact of PMV in the field of surgery for SM has not been analyzed to date. In the present study we therefore assessed the prognostic impact of PMV regarding early postoperative mortality and overall survival (OS) in patients who had undergone surgery for SM.

## Methods

### Patients and study design

All patients with SM aged > 18 years who had undergone primary posterior spinal canal decompression with or without instrumentation between 2015 and 2019 at the neurosurgical department of the University Hospital Bonn were entered into a computerized database (SPSS, version 25, IBM Corp., Armonk, NY). Follow-up checks were conducted after 3 and 12 months. Patients*’* clinical information including age, sex, primary tumor, location of SM, surgical procedure, number of affected vertebrae, ASA score, neurological and functional status (American Spinal Injury Association: ASIA Score (19)), and overall survival (OS) was recorded. The Karnofsky Performance Scale (KPS) was used to evaluate patients*’* preoperative functional status. We excluded all patients who were not classified as operable and those without complete data or follow-up information.

Indications for surgery as well as its extent were determined according to the Spinal Instability Neoplastic Score (SINS) (20, 21). Every patient received preoperative CT and MRI scans of the affected spinal level (22, 23). Patients with spine instability received posterior dorsal decompression with stabilization – because of pedicle system failure, pathological kyphosis of the spine, lytic bone lesions, or neurological deficits. Patients were treated by one of three neurosurgeons with many years*’* experience in spine surgery, all of whom used the same standardized workflow (including median posterior approach and navigation system) and the same instruments (Diplomat system, Signus Alzenau, Bavaria, Germany). Our standard surgical procedure consisted of the following steps: median posterior dorsal approach, open transpedicular screw implantation (we did not use a percutaneous system), spinal canal and nerve root decompression in combination with posterior bone fusion. We used Mastergraft Granules (Medtronic) rather than cages for the posterior fusion. During cervical and cervicothoracic instrumentation, we used only posterior fixation and dorsal fusion.

In cases of spine stability without pedicle failure or kyphosis and blastic bone lesions, the patients only received dorsal spinal canal and nerve root/spinal cord decompression *via* laminectomy of the affected segment(s) without stabilization. A biopsy from the tumor and bone was taken for histopathological analysis in each case, regardless of the surgical treatment. Patients with dorsal instrumentation received a CT scan immediately after the operation, which was used for comparison purposes in the follow-up checks. Patients who needed intensive medical monitoring were transferred to our intensive care unit, otherwise they received normal post-surgical care.

Once the results of the histopathological analysis were received, all cases were reviewed by our internal Neurooncological Tumor Board consisting of neurosurgeons, radiation therapists, neurooncologists and neuroradiologists. The recommendations for post-surgery management, such as further surgical treatment or other therapy options such as chemotherapy or radiation, were thus based on collective decision-making.

PMV was defined as an invasive ventilation period of > 24 hours after initial spinal surgery (16, 17, 24, 25). The comorbidity burden was measured using the Charlson comorbidity index (CCI) (26, 27).

Early postoperative complications were assessed using a publicly available list of adverse events introduced by the Agency for Healthcare Research and Quality and the Center for Medicare and Medicaid Services, and referred to as patient safety indicators (PSIs) and hospital-acquired conditions (HACs) (28–31). PSIs included acute myocardial infarction, pressure ulcers, iatrogenic pneumothorax, transfusion reactions, peri- and postoperative hemorrhage, pulmonary embolism, acute postoperative respiratory failure, deep vein thrombosis, postoperative sepsis, and wound dehiscence, as well as accidental puncture or laceration. Within the group of HACs, screening was performed for pneumonia, catheter-associated urinary tract infection, surgical site infection, blood incompatibility, crushing injury, manifestation of poor glycemic control (diabetic ketoacidosis, non-ketonic hyperosmolar coma, hyperglycemic coma), fall injury, and vascular catheter-associated infection. In addition, to assess complications specific to spinal surgery, postoperative records were screened for cerebrospinal fluid (CSF) leakage, postoperative meningitis, and implant failure, as well as postoperative new or worsened neurological deficits. These were classified as spinal surgery-related complications (SSCs). As described elsewhere, perioperative complications were defined as any postoperative adverse events, with or without further surgical intervention, occurring within 30 days of the initial surgery (32).

Overall survival (OS) was measured starting from the day of SM surgery until death or last observation. Patients for whom no further follow-up information was available (e.g. due to further treatment at external institutions) were excluded from further analysis. All parameters were compared in relation to OS.

This study was conducted in accordance with the 1964 Helsinki declaration and approved by the Ethics Committee of the University Hospital Bonn (protocol no. 067/21). Informed consent was not sought as a retrospective study design was used.

### Weaning protocol

Patients with prolonged oral intubation and ventilation underwent swiftly tracheotomy. After tracheotomy, weaning phase begins on our intensive care unit, first for hours, then weaning depending on the patient’s clinical condition. If patients continue to be ventilated, they will be transferd to a rehabilitation facility for further weaning, which has the option of combined rehabilitation and weaning. After the end of the weaning phase (3 weeks), patients were transferred to our clinic, for re-evaluation of clinical status. If they have recovered well, they will be treated further with chemotherapy and radiation therapy. If the clinical situation remains poor, they will be treated palliative.

### Statistics

Data analyses were performed using SPSS (version 25, IBM Corp., Armonk, NY) and PRISM computer software packages. Categorical variables were analyzed in contingency tables using Fisher’s exact test. The Mann-Whitney U test was chosen to compare continuous variables as the data were mostly not normally distributed, while non-parametric data are summarized by median values (first quartile – third quartile). Results with p < 0.05 were considered statistically significant. *Univariate analysis* (including following factors: primary tumor size, median age, gender, location and levels of disease, median CCI, perioperative neurological deficits, surgery, median duration of surgery, early postoperative complications, 30day/one year mortality and median OS) *was conducted using Fisher’s exact test (two-sided) and the independent t-test. P values <0.05 were considered statistically significant*. In addition, in order to determine independent predictors of 30 days mortality in patients with surgically-treated spinal metastasis, a backward stepwise method was used to construct a multivariate analysis using a binary logistic regression, again with p < 0.05 being considered statistically significant. Additionally, we decided to add a Cox regression analysis in order to identify factors that are significantly associated with worsened OS. Under consideration of known prognostic parameters (patient age, tumor entity, ASIA classification score value, preoperative KPS, number of affected spinal levels), a preoperative KPS > 70, lung cancer and PMV >24 hrs significantly correlated to shortened OS.

## Results

### Patient characteristics and demographic data

Between January 2015 and December 2019, 198 patients were surgically treated for SM at the authors*’* neurosurgical department. The median age was 66 years (range 57-74 years). The most common primary tumor site was the lung (23%), followed by the prostate (20%) and the breast (11%) (**Table 1**). The thoracic spine was the most commonly affected spinal section (56%). Single or dual-level disease was present in 120 of 198 patients (61%), whereas multilevel disease was present in 78 patients (39%). The majority of patients (63%) underwent decompression with additional dorsal instrumentation, while decompression alone was performed in 37% of cases. 126 of 198 patients (64%) presented with a preoperative KPS score of > 70.

**Table 1:** Patient characteristics*.

	n = 198
**Median age (IQR) (in yrs)**	66 (57–74)
**Female sex**	76 (38)
**Primary tumor site**	
** Lung**	46 (23)
** Breast**	22 (11)
** Prostate**	40 (20)
** Other**	90 (45)
**Location of disease**	
** Cervical**	20 (10)
** Thoracic**	111 (56)
** Lumbar**	33 (17)
** Combined**	34 (17)
**Surgery**	
** Decompression**	74 (37)
** Stabilization**	124 (63)
**Levels of disease**	
** 1-2**	120 (61)
** ≥ 3**	78 (39)
**Median CCI (IQR)**	8 (6-10)
**ASA score ≥ 3**	126 (64)
**KPS ≥ 70**	126 (64)
**Pre-operative neurological deficit**	46 (23)
**Median OS (IQR) (in months)**	11 (3-24)
**Postoperative PMV**	20 (10)

*Values represent the number of patients unless indicated otherwise (%).

ASA, American Society of Anesthesiology physical status classification system; ASIA, American Spinal Injury Association; CCI, Charlson comorbidity index; KPS Karnofsky Performance Scale; IQR, interquartile range; n, number of patients; OS, overall survival; PMV, prolonged mechanical ventilation; yrs, years.

Median OS for all patients with surgically treated SM was 11 months (interquartile range [IQR] 3-24). A total of 20 out of 198 patients (10%) underwent postoperative PMV. For further details of patients*’* and tumor-related characteristics see **Table 1**.

### Patient-related and disease-related factors associated with postoperative PMV

Out of 20 patients with postoperative PMV, 10 (50%) exhibited tumor-related preoperative neurological morbidity (ASIA A-C) compared to 36 of 178 patients (20%) without PMV (p = 0.01) (**Table 2**). At 240 minutes (IQR 170-294), the median duration of surgery for these patients was significantly longer than 178 minutes (IQR 125-244) for those without postoperative PMV (p = *0.03).*

**Table 2:** Factors associated with postoperative PMV following surgery for spinal metastasis*.

	Patients without PMV n = 178	Patients with PMV n = 20	p-value
**Median age (yrs)**	64 (56-76)	66 (57-74)	0.78
**Female sex**	68 (38)	8 (40)	1.0
**Primary tumor site**			
Lung	38 (21)	8 (42)	0.09
Breast	22 (12)	0 (0)	0.13
Prostate	39 (22)	1 (5)	0.08
Other	79 (44)	11 (55)	0.48
**Location of disease**			
Cervical	19 (11)	1 (5)	0.70
Thoracic	99 (56)	12 (60)	0.81
Lumbar	30 (17)	3 (15)	1.0
Combined	30 (17)	4 (20)	0.75
**Levels of disease**			0.15
1-2	111 (62)	9 (45)	
≥ 3	67 (38)	11 (55)	
**Median CCI (IQR)**	8.5 (7-10)	8 (6-10)	0.58
**Preoperative neurological deficit (ASIA Score)**			**0.0171**
A-B	23 (12.9)	7 (35)	
C-E	155 (87.1)	13 (65)	
**Surgery**			0.81
Decompression	66 (37)	8 (40)	
Stabilization	112 (63)	12 (60)	
**Median duration of surgery (IQR)**	178 (125-244)	240 (170-294)	**0.03**
**Early postoperative complications**			
PSIs	11 (6)	4 (20)	0.05
HACs	7 (4)	3 (15)	0.27
Specific SSCs	4 (2)	1 (5)	0.42
**30 day mortality**	9 (5)	14 (70)	**< 0.0001**
**1 year mortality**	85 (48)	20 (100)	**< 0.0001**
**Median OS (IQR)**	12 (4-26)	1 (0-7)	**< 0.0001**

*Values represent the number of patients unless indicated otherwise (%). Bold values means statistically significant.

CCI, Charlson comorbidity index; HAC, hospital-acquired conditions; IQR, interquartile range; OS, overall survival; PMV, prolonged mechanical ventilation; PSIs, patient safety indicators; SSCs, spinal surgery-related complications; yrs, years. Median (IQR).

Two out of 19 patients (11%) with PMV exhibited postoperative pulmonary embolism, 2 patients (11%) suffered from postoperative hemorrhage with indication for revision surgery, 3 patients revealed postoperative pneumonia (15%) with respiratory failure. Furthermore, 8 of 19 patients (42%) with postoperative PMV suffered from lung carcinoma therefore exhibiting elevated risk profiles for postoperative PMV as well as prolonged time of weaning.

Age, primary tumor site, the number of affected spinal levels, preoperative CCI, and peri- and postoperative complications, among others, did not significantly differ between the two groups of patients with and without PMV **(** **Table 2** **).** The postoperative complications were postoperative hemorrhage (2%), Postoperative pulmonary embolism/deep vein thrombosis (5%), Wound dehiscence (1%), Pneumonia (3%), Catheter-associated urinary tract infection (2%), and CSF leakage (3%).

**Figure 1:**
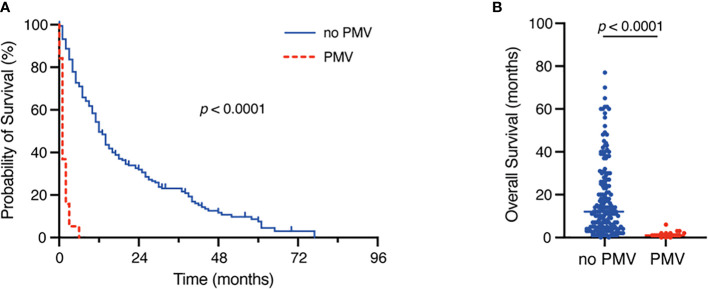
Kaplan-Meier survival analysis **(A)** and dot plots **(B)** dependent on the occurrence of postoperative PMV. PMV, prolonged mechanical ventilation.

A total of 14 out of 20 patients (70%) with PMV died within 30 days of surgery compared to 5 of 178 patients (9%) without PMV (p < 0.0001). Patients with PMV exhibited a median OS of 1 month (IQR 0-7 months) compared to 12 months (IQR 4-26 months) for patients without PMV (p < 0.0001) (**Table 2**, **Figure 1**).

### Multivariate analysis identifies PMV as an independent predictor of 30 days mortality

We conducted a multivariate analysis in order to identify independent pre- and perioperative predictors of 30 days mortality following surgery for spinal metastasis. The multivariate analysis identified *“*PMV > 24 hours*”* (p < 0.001, OR 0.3, 95% CI 0.02-0.4) as the only significant and independent predictor of 30 day mortality (Nagelkerke*’*s R^2^ 0.38).

**Figure 2:**
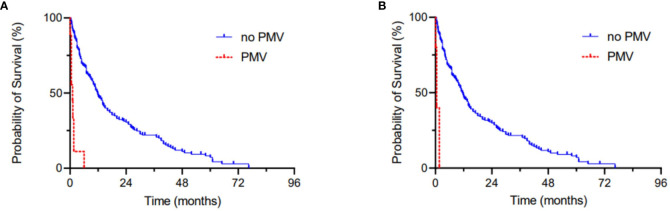
Kaplan-Meier survival analyses dependent on the occurrence of postoperative PMV > 48 hrs **(A)** and > 72 hrs **(B)**.

Ten of 198 patients (5%) exhibited postoperative PMV with a ventilation time of > 48 hrs., 5 of 198 patients (3%) exhibited postoperative PMV with a ventilation time of > 72 hrs. Kaplan-Meier survival analysis revealed a mOS of 1 month for the 48 hrs. cut off-value of PMV (p < 0.0001) and a mOS of 0.5 months for the 72 hrs. cut-off value of PMV (p < 0.0001) (**Figure 2**).

Furthermore, **Figure 2** was added depicting Kaplan-Meier survival analyses for the 48 hrs. and the 72 hrs. cut-off value for PMV

### Cox regression analysis

“Cox regression analysis under consideration of patient age, tumor entity, ASIA classification score value, preoperative KPS, number of affected spinal levels as known prognostic relevant parameters identified a preoperative KPS < 70 (Hazard ratio (HR) 0.3, p<0.001, tumor entity lung (HR 0.6, p=0.03’) and PMV >24 hrs (HR 0.15, p<0.001) as factors that were significantly associated with worsened OS”.

## Discussion

This study analyzes the prognostic impact of postoperative PMV in patients who had undergone surgical therapy for SM. We found that PMV was significantly correlated to high early postoperative mortality rates and poor OS.

Our results regarding the entity distributions reflect widely known study results (33–35). In our study, the thoracic spine was found to be the most frequently affected part of the spine in accordance with numerous studies (36, 37). A specific distribution pattern depending on the primary tumor, such as metastasis of lung cancer preferentially into the thoracic spine, could not be confirmed in some studies (38, 39). Contrary to this, other authors described bronchial carcinoma in the thoracic spine, prostate carcinoma as the most common primary in the lumbar spine (33). The literature describes multiple spinal metastasis up to 30% in the cases of SM, in our cohort we had 39% with multilevel SM (40). The gender distribution shows in our data as well as in the literature a male dominance (41, 42). One possible reason for this distribution may be that, prostate carcinoma is a common gender-specific tumor with high spinal metastasis tendency (43). Based on the KPI, the preoperative general condition of the patients was assessed, the score was 70% for the majority of our patients, in many studies the KPI varies between 50-70% (44, 45).

The optimal treatment of symptomatic SM is the focus of the therapy, and the aim of the several treatment options is usually limited to the maintenance or improvement of neurological function, reduction of pain, local tumor control, and improvement of the patient*’*s general quality of life (41, 46). Recently, several patient-related and disease-related characteristics have been reviewed for their prognostic value and summarized in the form of prognostic systems and parameters. All these studies are seeking to predict life expectancy as accurately as possible, so as to be able to recommend the most appropriate treatment for the patient (20, 41, 47–50). However, surgery for SM in this patient cohort may require prolonged postoperative intensive care, which may adversely affect the anticipated benefit of the surgery.

PMV has previously been identified as a meaningful prognostic factor in patients suffering from several tumor diseases (16, 51). Recently, PMV of more than 48 hours has been reported to result in median OS of < 1 month in patients with surgically treated brain metastasis (16), therefore indicating that PMV constitutes a devastating prognostic factor in neurosurgical oncology. Similarly, PMV of more than 24 hours has been identified as an independent prognostic factor in patients undergoing surgery for glioblastoma, with a reported median OS of as low as 3 months (18). In an analysis of 5,138 cases, Shish et al. reported the 1 year survival rate in patients with malignancies and in need of PMV to be as low as 14% (24). There has been no analysis to date of the subgroup of patients with SM. This subgroup poses an additional challenge as it comprises critically ill patients at an advanced stage of metastatic cancer disease. Furthermore, unlike this study, most currently available data on PMV in the field of cancer treatment do not specifically focus on the subgroup of patients who underwent oncological surgery. Not all patients with SM are treated surgically: particularly in the case of small, non-space occupying tumors of the spinal canal or multiple asymptomatic findings, other treatment options are well established (52–56). Along these lines, the subgroup of patients with SM and additional surgical treatment are supposed be at a high risk of postoperative PMV. Regardless of the reason for surgical treatment, surgery induces a significant degree of surgical trauma (57, 58). Postoperative PMV in patients with surgically treated SM may be necessary not only because of the patient*’*s weakness or a disease such as lung cancer, but also because of the localization of the SM surgery or because of associated postoperative complications (59–63). We found no correlation in our cohort between postoperative PMV and the primary tumor site, the spinal location affected, or the number of affected spinal levels. Instead, the group of PMV patients exhibited higher levels of preoperative neurological deficits and a significantly longer median surgery duration. These findings are in line with several reports linking postoperative PMV occurrence to elevated surgery duration and preoperative morbidity (64, 65). These findings point at recent efforts to use preoperative risk stratification to more comprehensively predict the course of early postoperative treatment (66). This study provides the only available data on PMV and prolonged intensive care in the field of surgery for SM. These data do not allow for preoperative risk assessment.

Furthermore, the unsatisfactory survival rates of patients with SM and postoperative PMV in the present study could also partly be attributed to a delay in postoperative adjuvant treatment and/or in further therapy for the underlying cancer disease (67, 68), the delay being caused by postoperative intensive care. Prolonged ICU observation of cancer patients and frequent communication with all clinical colleagues and with the patient or their authorized representative are important and indeed a basic aspect of interdisciplinary treatment. It is in the best interests of the patient for the neurosurgeons, neurooncologists and intensive care physicians to jointly determine the patient*’*s ICU therapy and decide on the next stage of treatment (69). Oncological re-evaluation of the patient*’*s prognosis after surgery and assessment of further treatment options can thus be complemented by the ICU physicians*’* knowledge of what is possible in the intensive care setting. Treatment providers should constantly check that continued treatment and an extended ICU stay are in accordance with the patient*’*s wishes.

The findings of this study should raise awareness of the small subgroup of cancer patients with high early postoperative mortality and a poor overall prognosis – that is, the subgroup of patients with surgically-treated SM who need postoperative PMV for more than 24 hours. Early pre-surgical stratification may help to identify patients who are at a high risk of prolonged postoperative intensive care treatment. Preoperative identification of these patients is a major challenge for future scientific endeavors due to the limited data available. It is nevertheless worthwhile in order to predict the most appropriate course of postoperative treatment and to inform communication with patients about what can realistically be expected from the neurosurgical procedure.

## Conclusions

Our data indicate postoperative PMV is significantly correlated to high early postoperative mortality rates as well as to poor OS in patients with surgically treated SM. The authors believe these findings justify further multicenter studies to comprehensively investigate PMV as an underestimated negative prognostic factor in the course of surgical treatment for SM.

## Limitations

The present study has several limitations. Acquisition of data was retrospective; data are therefore subject to well-known and well-described types of bias. Patients were not randomized and their treatment was decided on by the neurosurgeon. Given the low incidence of postoperative PMV occurrence, the number of patients with PMV is quite small, which means the univariate and multivariate analyses may be subject to error. The authors intend to consider these data as a first estimation of a potential correlation between postoperative PMV and worsened survival in patients with surgery for SM. This may lead to further investigations structured to avoid the potential selection bias due to the limited group size in this study.

Additionally, in regard of the small patient cohort of 19 patients with PMV > 24 hrs, the present study did not allow for cut-off value determination in order to specifically identify the time of postoperative mechanical ventilation leading to an increase in early postopoerative mortality and reduced survival. Further multicenter studies will be needed in order to sufficiently address this issue.

## Data availability statement

The original contributions presented in the study are included in the article/supplementary material. Further inquiries can be directed to the corresponding author.

## Ethics statement

All of the procedures performed were in line with the ethical standards of our institutional and national research committee (Ethics committee of the Rheinische Friedrich Wilhelms University Bonn) and with the 1964 Helsinki declaration and its later amendments or comparable ethical standards. The local ethics committee at the University of Bonn approved this study (protocol no. 067/21).

## Author contributions

Conceptualization, MH and MB. Methodology, MH and MB. Software, A-LP. Validation, MH, MS and MB. Formal analysis, MS and A-LP. Investigation, SB, SR, LE, CB, FS, AR, NS, UH, MK, FG, HV. Resources, HV. Data curation, SB, SR and MB. Writing—original draft preparation, MH, MS and MB. Writing—review and editing, all authors. Visualization, MH, MS and MB. Supervision, MB and MS. Project administration, MS, MB. All authors contributed to the article and approved the submitted version.

## Conflict of interest

The authors declare that the research was conducted in the absence of any commercial or financial relationships that could be construed as a potential conflict of interest.

## Publisher’s note

All claims expressed in this article are solely those of the authors and do not necessarily represent those of their affiliated organizations, or those of the publisher, the editors and the reviewers. Any product that may be evaluated in this article, or claim that may be made by its manufacturer, is not guaranteed or endorsed by the publisher.

## Abbreviations

ASA, American Society of Anesthesiology; ASIA Score, American Spinal Injury Association; CCI, Charlson comorbidity index; CI, Confidence interval; CT, Computer tomography; HACs, Hospital-acquired conditions; IQR, Interquartile range; KPI, Karnofsky Performance Scale; MRI, Magnetic resonance imaging; OR, Odds ratio; OS, Overall survival; PMV, Prolonged mechanical ventilation; PSIs, Patient safety indicators; SM, Spinal metastasis; SSCs, Spinal surgery-related complications.
